# Trends in vital signs and routine biomarkers in sepsis patients during resuscitation in the emergency department: a prospective observational pilot study

**DOI:** 10.1186/2197-425X-3-S1-A301

**Published:** 2015-10-01

**Authors:** VM Quinten, M Van Meurs, JC Ter Maaten, JJM Ligtenberg

**Affiliations:** Emergency Department, University Medical Center Groningen, Groningen, Netherlands; Department of Critical Care, University Medical Center Groningen, Groningen, Netherlands; Department of Pathology and Medical Biology, Medical Biology Section, University Medical Center Groningen, Groningen, Netherlands

## Introduction

Sepsis lacks a reliable measure of disease activity [1, 2]. Therefore, it remains unclear how to monitor the response to treatment. Little is known about changes in vital signs during sepsis resuscitation and biomarkers for disease activity are not available to evaluate effects of treatment in sepsis patients at the bedside [2, 3]. Trends in vital signs and biomarker levels during resuscitation might provide information about response to treatment at a very early stage of sepsis.

## Objectives

Detect trends in vital signs and routine biomarker levels during sepsis resuscitation in the emergency department (ED).

## Methods

Prospective observational pilot study in the ED of a tertiary care teaching hospital. Adult non-trauma patients with two or more SIRS criteria and suspected infection were included. Blood samples were taken and vital signs (heart rate, blood pressure, respiratory rate, oxygen saturation and temperature) measured at admittance to the ED (T_0_) and 3 hours later (T_1_) and the differences between T0 and T3 (delta) were analyzed.

## Results

**Figure 1:**
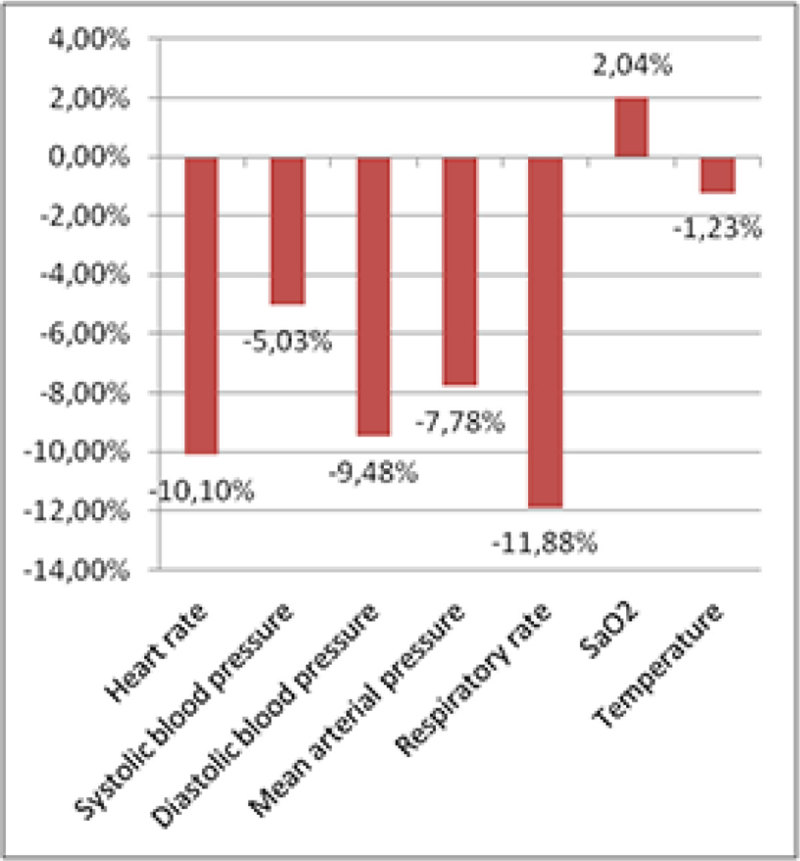
**Delta in vital signs between T**_**0**_ **and T**_**1**_ **.**

**Figure 2:**
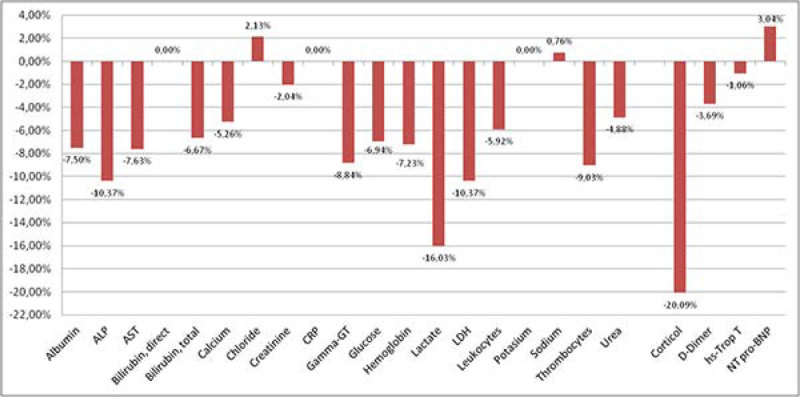
**Delta in biomarker levels between T**_**0**_ **and T**_**1**_ **.**

In total data of 99 patients was analyzed. Of these patients, 63 presented with sepsis, 30 with severe sepsis and 6 patients had septic shock. Trends in vital signs and routine biomarker levels are respectively shown in Figure 1 and Figure 2. All vital signs decreased, except for peripheral oxygen saturation which increased. The heart rate and respiratory rate dropped by over 10% during resuscitation (p < 0.001). At the same time, the systolic and diastolic blood pressure decreased respectively with 5% and more than 9% (p < 0.001). Almost all biomarker levels decreased during resuscitation, except for CRP, bands, potassium, Troponin T and direct bilirubin that remained stable. Sodium, chloride and NT pro-BNP increased slightly.

## Conclusions

Vital signs and biomarker levels showed descending trends during resuscitation, except for parameters directly affected by treatment modalities. Despite these trends patients clinically improved. Trends in vital signs and routine biomarkers might be helpful in predicting clinical course and response to treatment in sepsis patients.
